# Health Sciences Students’ Self-Assessment of Information and Communication Technology Skills and Attitude Toward e-Learning

**DOI:** 10.2196/mededu.5606

**Published:** 2016-06-20

**Authors:** Ali Jassem Buabbas, Hamza Mohammad Hassan Al-Shawaf, Abdullah Abdulaziz Almajran

**Affiliations:** ^1^ Faculty of Medicine Department of Community Medicine and Behavioral Sciences Kuwait University Jabriya Kuwait; ^2^ Faculty of Allied Health Sciences Department of Health Information Administration Kuwait University Jabriya Kuwait

**Keywords:** ICT literacy, health sciences student attitudes, health informatics, e-learning, medical education

## Abstract

**Background:**

In medical education, information and communication technology (ICT) knowledge and skills have become a necessity and an integral part of preparing tomorrow’s doctors to be sufficiently competent to use informatics resources effectively and efficiently for the best practice of medicine.

**Objective:**

This research aimed to study the literacy of the preprofessional students in ICT before and after taking the basic informatics course at the Health Sciences Center at Kuwait University, to understand their potential and their attitudes toward using ICT, including e-learning.

**Methods:**

A validated questionnaire was used to collect data from 200 students in 2 stages: before and after the informatics course on the preprofessional program. In addition, the tutors’ observational assessments of the students’ achievements during the informatics course were obtained.

**Results:**

The response rate of students before the course was 85.5% (171/200) and after was 77% (154/200). Of 200 students, 85% were female, and 15% were male. This disproportional representation of genders was due to the fact that 85% of registered students were female. Approximately 59% (101/171) of the students assessed themselves before the course as computer literate; afterward, this increased to 70.1% (108/154). Students who were still computer illiterate (29.2%; 45/154) mostly used the excuse of a lack of time (60%; 27/45). In generic ICT skills, the highest levels were for word processing, email, and Web browsing, whereas the lowest levels were for spreadsheets and database. In specific ICT skills, most respondents were reported low levels for statistical package use and Web page design. The results found that there was a significant improvement between students’ general ICT skills before and after the course. The results showed that there were significant improvement between how frequently students were using Medline (*P*<.001), Google Scholar (*P*<.001), and Cochrane Library (*P*<.001) before and after the informatics course. Furthermore, most of the students who completed the course (72.8%; 110/151) chose the learning management system as the most useful e-learning tool. The results of the tutors’ assessments confirmed the obvious improvement in most of the students’ skills in using ICT.

**Conclusions:**

The ICT knowledge and skills of the students before the course seemed insufficient, and the magnitude of the improvements that were acquired throughout the informatics course was obvious in most of the students’ performance. However, the findings reveal that more practice was required. The attitudes of most of the students toward the potential of e-learning were considered positive, although the potential of Web-based learning in medical training was not well known among the students.

## Introduction

In medical education, information and communication technology (ICT) knowledge and skills have become a necessity and an integral part of preparing tomorrow’s doctors to be sufficiently competent to use the varied informatics resources effectively and efficiently for the best practice of medicine. Introducing ICT at early stages in the medical education system will ensure that future health care providers are well equipped and able to use different informatics resources in an effective and efficient manner to improve their practices. For instance, ICT enhances competency in the use of medical databases and search strategies, the navigation of websites, the use of Microsoft Office, and the use of e-learning, as well as the use of electronic records [1,2].

Many higher education institutions around the world have adopted ICT to enhance the educational process. Incorporating ICT into education is widely adopted by developed countries, in which the Internet, Web-based databases, e-learning, and other resources can be used by the students and staff [3]. In the United States, the need to introduce ICT into medical education was demonstrated when academic staff found themselves responsible for a wide array of current topics that needed to be delivered to the students [4]. However, the standard educational method alone could not support such needs at that time. Furthermore, countries in the Middle East, such as Egypt, Jordan, and Saudi Arabia, have implemented different ICT tools, which have improved the students’ leaning experience [5-8]. In Iran, the impact of digital technologies applications on education has been studied [9,10], with particular attention on ICT literacy of medical students [11,12]. In Kuwait, research studies at Kuwait University found into information literacy and Internet use among students [13] and effectiveness of e-learning on learning environment [14].

### Kuwait University Health Sciences Center

Kuwait University has experience of adopting and using ICT to support the traditional teaching and learning processes, but few of the faculties have used these facilities. The matter is not about bringing technology to the educational field, it is about the competence of the user or student that is going to use the computer technology efficiently.

The Health Sciences Center (HSC) at Kuwait University uses only a conventional educational method, which focuses on the material itself rather than the learner, although the facilities are equipped with modern computer systems, computer laboratories, and a library supported by varied e-resources. Kuwait University is striving toward attaining the optimum environment for students to acquire the best education that keeps pace with developments worldwide by availing the latest technologies. As a result, an informatics course in a preprofessional program has been prepared for all students from medicine, dentistry, and pharmacy who have newly joined the HSC.

In many universities, obligatory courses in basic computer knowledge are offered to students, although there are still differences in computer skills among the students [2]. In Kuwaiti society, the use of ICT is widespread and has spread among people through mobile phones and notebooks. In addition, their experiences in using computers made us curious to know what ICT skills the students had before joining the university.

Overall, in the Middle East, little research has been done on ICT or informatics in medical education [5,8]. Consequently, assessing students for their literacy in using ICT at the HSC is considered new research at Kuwait University.

### Research Questions

- Are preprofessional students literate in using ICT for faculty-related purposes at the HSC?
- What are the attitudes of the students toward using an e-learning system at the HSC?

This research aimed to study the literacy of the preprofessional students in ICT before and after taking the basic informatics course at the HSC at Kuwait University, to understand their potential and their attitudes toward using ICT, including e-learning.

### Research Objectives

Before starting the course:

1. To explore the existing ICT knowledge and skills of the preprofessional students.

2. To explore the attitudes of the students toward using the e-learning system.

After completing the course:

1. To identify the ICT knowledge and skills acquired after completing the informatics course.

2. To assess the attitudes of the students toward using the e-learning system.

## Methods

### Study Design and Population

This cross-sectional study enrolled first-year students attending the HSC at Kuwait University who were registered on the “Informatics in Healthcare” course. This course is mandatory for all preprofessional students from medicine, dentistry, and pharmacy in the first semester at the HSC in Kuwait University. It covers the following topics: an overview of health informatics, the information hierarchy of the health information system, Web-based medical resources, and medical information retrieval, in addition to the use of Microsoft Office programs, which are offered over the Internet to the students via the electronic resources of Kuwait University. This course on informatics in health care is taught once a week and is supported by computer laboratory sessions, where students can practice accessing Web-based databases to locate specific research articles and to evaluate formal website design using specific criteria. Informed consent was obtained from each participant in the study. Ethical approval was elicited from the Research Committee at the HSC at Kuwait University.

### Study Design

This study used an ICT literacy and resource utilization survey, which is a validated questionnaire [15]. This questionnaire was modified to suit our research objectives. This modification included excluding some ICT skills from the questionnaire, which encompassed: 2 generic ICT skills (programming and software installation) and 7 specific ICT skills, namely: print out a document, cut and paste information, draw and paint, organize, learn new applications, play games, and set up a mailbox. These excluded items were found to be irrelevant to the health informatics course, although some specific skills were asked in the questionnaire in a general form, such as print out a document, cut and paste information, and set up a mailbox. The questionnaire was pretested with 20 students, who were excluded from the main study.

The collection of data was performed in 2 stages. Stage 1: Before starting the course in September 2015, the students were asked in the introductory lecture to complete a Web-based questionnaire that was posted on “Moodle” (the Web-based learning environment). Stage 2: After completing the course in December 2015, the students were asked to complete the same Web-based questionnaire. The ICT literacy of the students was assessed using a questionnaire with 20 items comprising 4 criteria: (1) demographic data; (2) ICT training and skills, including (a) generic ICT skills and (b) specific ICT skills; (3) ICT resource availability and utilization; and (4) attitudes toward e-learning using computer skills.

In addition to the self-perceived questionnaire, an assessment was included in this study to maximize the validity of the individualized assessment. This was made through sending an email to 3 of the course tutors to obtain their opinions on students’ achievements during the course. This assessment was based on the students’ competencies, including course assessments in the following items: (1) skills in using computer software, such as Microsoft Office, including word processing, where the students were assessed in creating and editing a document, writing 100 words, inserting a table using given data, and inserting a page number, header, and footer. In regard to Excel sheets, the students were assessed in opening a sheet, inserting a table with given data, using a formula to calculate the average percentage, inserting a column with a title, and using ascending and descending orders. In regard to PowerPoint graphical presentations, the students were assessed in creating a slide with a title, inserting a table using given data, using a chart to present the data graphically, inserting and resizing the chart image, and changing the font size. Finally, all files had to be given new names and saved to the desktop. (2) Using different electronic resources to retrieve medical information and research articles, the students were asked a question that required access to the e-library at Kuwait University to retrieve medical information, such as a description of a disease or a clinical use of a medication. Moreover, a citation was provided to the students to search and find the full-text article using the PubMed, Scopus, or Ovid databases. Also, the tutors were asked to describe (3) the students’ attitudes regarding using “Moodle” as a learning management system with multiple functions, such as Web-based discussions.

### Statistical Analysis

For the questionnaire, the data obtained were processed and analyzed using the Statistical Package for the Social Sciences (SPSS version 23). Comparisons of frequencies before and after the course were performed using the chi-square test, considering the probability value *P*<.05 as statistically significant. For the data obtained from the tutors, analysis was made manually using the aforementioned 3 items of course assessment to assess the students’ achievements appropriately.

## Results

### Questionnaires

#### Description of Study Population

Of 200 students, 171 responded to the questionnaire before starting the course, giving a response rate of 85.5%. By contrast, 154 students completed the questionnaire after completing the course, with a response rate of 77%.

#### Demographic Data

Table 1 summarizes the characteristics of the sample study. Most students were female, representing 84.9% (276/325) of the total respondents. This disproportional representation of genders was due to the fact that 85% of the registered students were female. Kuwaiti students represented 90.2% (293/325) of the total respondents, which constituted most of the sample.

**Table 1:** Demographics of the preprofessional students.

Characteristics		Total No. (%)	Before No. (%)	After No. (%)
**Number**		325	171	154
**Age (Mean±SD, years)**		17.8 **±**0.60	17.7 **±**0.62	17.9 **±**0.57
	17	96 (29.7)	62 (36.5)	(22.2)
	18	193 (59.8)	92 (54.1)	101 (66)
	19	34 (10.5)	16 (9.4)	18 (11.8)
**Gender**				
	Female	276 (84.9)	145 (84.8)	131 (85.1)
	Male	49 (15.1)	26 (15.2)	23 (14.9)
**Nationality**				
	Kuwaiti	293 (90.2)	152 (88.9)	141 (91.6)
	Non-Kuwaiti	32 (9.8)	19 (11.1)	13 (8.4)

#### ICT Training and Skills

The students were asked 4 questions; 3 of them were about their background in using ICT, including queries about previous training courses, computer literacy, and reasons for illiteracy. The last question of this section assessed the students’ ICT skills, which were divided into 2 levels: generic and specific items (see Table 2). Each item was rated on a scale of 1 to 4 regarding the proficiency of use (“I do not know,” “some elementary skills,” “can use it but need to learn more,” and “acceptable skills,” which means that the student feels at ease when using computer software).

**Table 2:** Generic and specific ICT skills.

Skill	Included items
Generic ICT skills	Use of word processing, Windows, file management, graphical presentations and PowerPoint, spreadsheets, email, Web-based databases, and Web browsing.
Specific ICT skills	Use of statistical packages, Web-based discussions, and Web page design.

The results of the questionnaires show that most of the students (63.9%; 108/169) had not received any previous ICT training before joining the university, whereas only 36.1% (61/169) of the students had received training on ICT, mostly in an informal way (72%; 44/61). The study shows that 60.1% (101/168) of the students assessed themselves as computer literate before taking the course, whereas 39.9% did not, mostly using the excuses of a lack of time (39%; 26/67) and not being interested in computers (37%; 25/67). After the students had completed the course, the results show that 70.6% (108/153) of the students assessed themselves as computer literate, whereas 29.4% (45/153) did not, citing lack of time (60%; 27/45) as the main reason.

#### Generic ICT Skills

In this section, the students were asked about their general ICT skills. Table 3 summarizes the self-assessment of students’ ICT knowledge and skills before and after the informatics course. The highest levels of students' skills were for word processing, email, and Web browsing, whereas the lowest levels were for spreadsheets and database. The study results before the course reveal that the students skills were mostly between the average (can use it but want to learn more) and acceptable levels (feel at ease when using computer software) for word processing, Windows, PowerPoint and graphical presentations, email, and Web browsing. Regarding other skills, including using spreadsheets and databases, most of the students were between the categories “no elementary skills” and “some elementary skills.”

After the health informatics course, most of the students’ generic skills were improved. The study results show that there were significant positive improvements in the students’ general ICT skills before and after the course in regard to the use of graphical presentations and PowerPoint (*P*=.001), spreadsheets (*P*=.006), Web-based databases (*P*<.001), file management (*P*=.002), and email (*P*=.046). By contrast, there were no significant improvements in the levels of students’ skills in regard to word processing (*P*=.290), Windows (*P*=.211), and Web browsing (*P*=.821) before and after the informatics course.

**Table 3:** Data analysis of the preprofessional students’ general ICT skills.

Skills		Before No. (%)	After No. (%)
**Word processing**			
	Do not know how to use it	12 (7.2)	9 (5.8)
	Some elementary skills	14 (8.4)	18 (11.7)
	Can use it but want to learn more	61 (36.7)	36 (23.4)
	My skills are acceptable	79 (47.6)	91 (59.1)
**Windows**			
	Do not know how to use it	7 (4.2)	3 (1.9)
	Some elementary skills	29 (17.3)	35 (22.7)
	Can use it but want to learn more	80 (47.6)	49 (31.8)
	My skills are acceptable	52 (31)	67 (43.5)
**File management**			
	Do not know how to use it	26 (15.5)	10 (6.5)
	Some elementary skills	48 (28.6)	34 (22.2)
	Can use it but want to learn more	54 (32.1)	58 (37.9)
	My skills are acceptable	40 (23.8)	51 (33.3)
**Graphical presentations and PowerPoint**			
	Do not know how to use it	17 (10.1)	9 (5.9)
	Some elementary skills	30 (17.9)	19 (12.5)
	Can use it but want to learn more	73 (43.5)	50 (32.9)
	My skills are acceptable	48 (28.6)	74 (48.7)
**Spreadsheet**			
	Do not know how to use it	60 (36.6)	40 (26.7)
	Some elementary skills	60 (36.6)	47 (31.3)
	Can use it but want to learn more	28 (17.1)	39 (26)
	My skills are acceptable	16 (9.8)	24 (16)
**Database**			
	Do not know how to use it	66 (39.5)	36 (23.5)
	Some elementary skills	54 (32.3)	36 (23.5)
	Can use it but want to learn more	38 (22.8)	44 (28.8)
	My skills are acceptable	9 (5.4)	37 (24.2)
**Email**			
	Do not know how to use it	6 (3.6)	3 (2)
	Some elementary skills	20 (12)	12 (7.9)
	Can use it but want to learn more	44 (26.3)	33 (21.7)
	My skills are acceptable	97 (58.1)	104 (68.4)
**Web browsing**			
	Do not know how to use it	6 (3.6)	3 (1.9)
	Some elementary skills	8 (4.8)	16 (10.4)
	Can use it but want to learn more	37 (22)	27 (17.5)
	My skills are acceptable	117 (69.6)	108 (70.1)

#### Specific ICT Skills

In this section, students were assessed regarding their specific ICT knowledge and skills before and after the informatics course. Before the course, the study results show that most of the students were between the average (can use it but want to learn more) and acceptable levels (feel at ease when using computer software) regarding Web-based discussions. Regarding other skills, such as Web page design and using statistical packages, most of the students were reported low levels, which fall the categories “no elementary skills” and “some elementary skills” (see Table 4).

From the study results, it is revealed that there were significant positive improvements between the students’ skills before and after the informatics course for statistical package use (*P*<.001) and Web page design (*P*<.001). However, there was no significant association with Web-based discussions, where *P*=.480, showing no significant improvement in the students’ skills.

**Table 4:** Data analysis of the preprofessional students’ specific ICT skills.

Skills		Before No. (%)	After No. (%)
**Use of a statistical package**				
	Do not know how to use it	55 (33.7)	31 (20.8)
	Some elementary skills	62 (38)	34 (22.8)
	Can use it but want to learn more	34 (20.9)	44 (29.5)
	My skills are acceptable	12 (7.4)	40 (26.8)
**Web-based discussions**				
	Do not know how to use it	26 (15.8)	26 (17)
	Some elementary skills	40 (24.2)	29 (19)
	Can use it but want to learn more	44 (26.7)	38 (24.8)
	My skills are acceptable	55 (33.3)	60 (39.2)
**Web page design**				
	Do not know how to use it	61 (36.7)	33 (21.9)
	Some elementary skills	64 (38.6)	50 (33.1)
	Can use it but want to learn more	32 (19.3)	30 (19.9)
	My skills are acceptable	9 (5.4)	38 (25.2)

#### ICT Resource Availability and Utilization

Ten questions were asked to the students about their main access to a computer or the Internet and how they use ICT resources (Microsoft Word, spreadsheets, charts, Web-based discussions, and searching the Internet for medical information), e-information sources, and e-learning programs, based on a frequency scale (“never,” “rarely,” “quite often,” and “very often”).

The results show that most of the students before (82.1%; 138/168) and after the study (81.5%; 123/151) had personal computers, which the majority had been using for 1 to 3 years. Moreover, a high percentage of the students were using family Internet connections, including a telephone line or cable or another type of broadband. From the overall results, it is reported that the students were spending (mean ± standard deviation) 7.7 ± 12.8 hours per week regularly doing their work on computers.

In regard to ICT activities, the results show that there were no significant improvements between students using ICT for studying (*P*=.162) or for leisure (*P*=.062) purposes, before and after the informatics course, using frequency scales, where *P*>.05. There was only a significant positive association that proved an increase in the research activity at the end of the course, where *P*=.002.

With respect to the utilization of ICT resources among the students, it was found that there were no improvements between the frequency levels of students before and after the course in using Microsoft Word (*P*=.088), using spreadsheets (*P*=.086), using charts (*P*=.414), using Web-based discussion boards (*P*=.148), and searching the Internet for medical information (*P*=.685). There was only a positive significant association with email use (*P*<.001), wherein most of the students (before: 34.5%; 59/171, after: 63.6%; 98/154) showed an increase in using email very often (more than 2 times a week). From the results, it was noticed that many of the students had never used spreadsheets (47.4%; 81/171) and charts (38%; 65/154) before the course.

In regard to using e-information sources, the results show that there were significant positive associations between how frequently students used Medline (*P*<.001), Google Scholar (*P*=.001), and Cochrane Library (*P*<.001) before and after the informatics course, showing an increase in the use of e-information sources **(**see Table 5).

Table 5 shows that there was an obvious change in the number of students who had never used Medline (before: 117, after: 53) or Cochrane Library (before: 123, after: 75).

**Table 5:** Data analysis of the preprofessional students’ use of e-information sources.

E-information sources		Before No. (%)	After No. (%)
**Medline (PubMed or Ovid)**				
	0 (Never)	117 (68.4)	53 (34.4)
	1 (Rarely: 1-7 times/semester)	37 (21.6)	64 (41.6)
	2 (Quiet often: 2-7 times/month)	7 (4.1)	27 (17.5)
	3 (very often ≥2 times/week)	1 (0.6)	6 (3.9)
**Google Scholar**				
	0 (Never)	58 (33.9)	37 (24)
	1 (Rarely: 1-7 times/semester)	35 (20.5)	61 (39.6)
	2 (Quiet often: 2-7 times/month)	25 (14.6)	29 (18.8)
	3 (very often ≥2 times/week)	44 (25.7)	22 (14.3)
**Cochrane Library**				
	0 (Never)	123 (71.9)	75 (48.7)
	1 (Rarely: 1-7 times/semester)	28 (16.4)	51 (33.1)
	2 (Quiet often: 2-7 times/month)	9 (5.3)	18 (11.7)
	3 (very often ≥2 times/week)	0 (0)	4 (2.6)

In the last question of this section, students were asked about their experience in using e-learning programs. The results showed that most of the students had experience with Web-based quizzes (before: 67.2%, after: 33.1%), image repositories (before: 16.3%, after: 9%), and learning management systems (before: 45%, after: 70.1%).

#### Attitudes Toward Using e-Learning

Two questions were asked to the students under this topic: question 1, with multiple choices about the most useful tools of e-learning from the students’ points of view; and question 2, which provided 3 options (agreement, neutral, and disagreement) and asked about specific statements regarding e-learning in medical education.

From the results, the students said that the learning management system is the most useful tool of e-learning, as it is a portal for Web-based courses, scoring 49.1% (83/169) before the course and 72.8% (110/151) after the course. In regard to using e-learning in medical education, specifically regarding disagreement with the notion that e-learning can replace lectures, significant results were reported before (102; 60.7%) and after the course (51; 33.1%), giving *P*<.001. Furthermore, the results show that most of the students disagreed (86 (50.5%), 95(61.6%)) that there was no need for e-learning programs for medical training (*P*=.07). Most of the students (124 (72.5%), 115 (74.6%)) said that e-learning systems (including Web-based training) should supplement lectures and exercises (*P*=.32). Moreover, the results show that most of the students (134; 78.3%) believed before the course that e-learning was nothing more than the distribution of notes over the Internet. This belief was abated after completing the course (71; 46.4%), giving *P*<.001.

### Tutors’ Assessment of Students’ ICT Skills

The results reveal that all 3 tutors confirmed that initially, most of the students had shown poor computer skills, such as using Microsoft Office. However, the students did well in the assessment after the course. As one of the tutors said, “ *most of the students were struggling at first in using computer skills* *; thereafter, they showed their enthusiasm and did well in the assessment.* ” Furthermore, the tutors stated that the students showed an obvious improvement in using numerous databases (PubMed, Google Scholar, Cochrane, Scopus, Ovid, and so forth) and became familiar with extracting medical information and using citations to retrieve full-text articles efficiently. Another tutor demonstrated that most of the students had become competent in using ICT for educational purposes through showing the total average grades of the students, which were 85% (very good) for using computer software programs and 92% (excellent) for using e-information resources and retrieving information or articles. In regard to the students’ attitudes toward e-learning systems, the tutors observed that most of the students found it easy to follow the e-learning site and made use of this facility effectively through using e-books, e-lecture notes, and materials, in addition to the Web-based discussion.

## Discussion

In this study, the literacy of the students, who came from different backgrounds, in ICT use was assessed, in addition to their attitudes toward using e-learning systems, which was of no lesser importance.

In general, the findings reveal that the informatics course made obvious improvements to the students’ knowledge and skills, as most of students' responses showed a willingness to learn and practice to get to an acceptable level in which they feel at ease using computer programs. Furthermore, most of the students agreed with using e-learning systems as a supplement to conventional learning, although the findings reveal a lack of knowledge on the potential of Web-based technologies in education.

The findings show that the number of male students was lower compared with the number of female students at the HSC, at a ratio of 1:3. This disproportional representation of genders was due to the fact that 85% (276/325) of registered students were female.

### The ICT Experience of Preprofessional Students at the HSC

The findings show that, before the course, more than half of the students assessed themselves as computer literate (60.1%; 101/168). After the informatics course had been completed in the preprofessional year, students' ICT literacy improved to 70.6% (108/153), whereas fewer students reported computer illiteracy. This could be explained that some of the students were occupied with their personal perceptions and expectations on the concept of literacy.

The findings of the study indicate that the students who were not interested (37%; 25/67) in computers, together with those who said they lacked time (60%; 27/45), lacked awareness about the potential impact of computers in education, particularly in medicine. Alternatively, it could be that using computers in education was not one of the top priorities of some of the students, as most of the students had received their ICT training informally (72%; 44/61) through private home sessions or from family or friends.

The findings reveal that the general ICT skills of the students improved after completing the course on informatics, especially regarding rating their use of Web-based literature databases as “acceptable” (before: 5.4%; 9/167, after: 24.2%; 38/154). Web-browsing skills had the highest percentage of students rating their skills as “acceptable”: 70% before and after the course. This could indicate that Web browsing is a common skill among people who use computer technology. This result is consistent with those of previous studies that found Web browsing is on top priority when searching for information [11,12]. The results of the tutors’ assessments of the students further complemented these findings and confirmed the competency of most of the students (average grade=85%) in using computer programs (Microsoft Word, Excel, and PowerPoint). Generally, this noticeable improvement in ICT skills pertained to the informatics course and its objectives, as it was intended to enable the students to use the e-resources offered by the HSC library and the e-learning center at Kuwait University.

On the basis of the findings of this study, the percentage of students who said they needed more knowledge in general ICT skills before the course on informatics was lower than the percentage who said they required it after the course. The impact of the informatics course on the students’ pattern of general ICT use was apparent. Nevertheless, the findings indicate that additional general ICT knowledge was still required, particularly on spreadsheets or Excel.

In regard to specific ICT skills, the findings demonstrate that approximately half of the students who had some basic skills in Web page design, Web-based discussions, and statistical packages still asked for more learning, particularly for statistical packages. These findings are comparable to results of previous studies [7,12] that found software, such as Excel and statistical packages were less familiar among students, in which a training program is recommended.

### The Preprofessional Students and ICT Resource Availability and Utilization

From the findings, it was revealed that most of the students (82% (before 138/168; after 123/151)) had personal computers with Internet connections. This indicates that computer and Internet connection availability was not an obstacle, particularly as these were offered by Kuwait University as well. These findings are consistent with those of a recent study in Saudi Arabia and another previous study in Jordan [8,5]. By contrast, in Egypt, some students (24.9%) found that having a computer with an Internet connection was a financial burden [7]. Furthermore, the frequency of email use among the students improved: before the course, 34.5% (59/171) of the students used email very often—this increased to 63.6% (98/154) after the course. A study in Kuwait University showed better results in regard to email use among social sciences students (70%) [13], in addition to other previous studies in the Gulf region, such as in Iran (86.8%) [6] and Saudi Arabia (98.6%) [8]. It seems that not all the preprofessional students were using email as the formal communication medium at the university, so they might have still been looking for person-to-person meetings, as they experienced when they were in high school.

After completing the course, improvement in the students’ activities using ICT was reported mostly for research purposes. This is consistent with another finding in this study, which indicates an obvious increase in the use of the Medline (PubMed or Ovid) and Cochrane databases rather than Google Scholar after the course on informatics. This indicates that the students improved their knowledge during the course regarding the retrieval of medical information or searching for full-text articles and became familiar with several medical databases, such as Medline. This finding is similar to that of another study [5,16], in which PubMed was the most Internet sites the medical students used to access for academic purpose. However, some other studies [8,17] showed that Yahoo and Google were on the top of the Internet sites for searching the Internet among medical students.

### Attitudes of Students Toward Using e-Learning in Medical Education

The findings show that the learning management system was chosen as the most useful tool of e-learning (before: 49.1%; 83/169, after: 72.8%; 110/151). This could demonstrate that the experience of the students in using “Moodle” throughout the course had an influence on their responses. In addition, some of the students preferred the easy way of getting material over the Internet at any time, as e-learning is considered a medium for sharing knowledge and experiences among students and staff, as shown by previous studies [18,19]. Moreover, the tutors of the course had observed most of the students enjoying e-learning and using the easy sharing of e-materials and knowledge through Web-based discussions.

Furthermore, the findings show that the students mostly disagreed (60.7%; 102/168) with the statement “Web-based learning programs are able to replace lectures” before the course started. The percentage of students who disagreed reduced to 33.1% (51/154) after the course, with an increase in agreement to 51.2% (79/154). This positive improvement could have resulted from the positive experience of students in using the Web-based learning management system during the course. However, the students preferred the blended learning system, reinforcing the findings of other studies [20-22]. Moreover, the findings show that the use of “Moodle” resulted in more positive responses from the students, which offered the students more individualization in the way of studying, as it supplemented the conventional learning approach. Hence, most of the students before (124; 72.5%) and after the study (115; 74.6%) preferred computer- and Web-based programs to support the traditional learning environment.

Finally, in regard to medical training and Web-based programs, 61.6% (95/154) of the students’ responses were negative, stating that they did not need these programs during medical training. This could indicate that most of the students were not knowledgeable about the potential of computer- and Web-based programs, such as the use of e-learning systems, to track training performance and to supply the trainers with up-to-date information. This reflects the need for an improvement in the knowledge of the potential and power of Web-based learning programs in medical training and education.

### Limitations

This study has some limitations: (1) the study excluded upper-class students in the faculties of medicine, dentistry, and pharmacy; (2) the study excluded other faculties of the HSC, such as allied health and public health; and (3) the students’ attitudes regarding e-learning were assessed using a limited number of variables. Hence, future research should be entirely focused on this topic, considering more variables [18]. Furthermore, due to the high prevalence of mobile technology (e.g, mobile phones) among students in Kuwait, future research would be recommended to study the use of mobile phone technology in medical education among students and academic staff, as these digital technologies require skills to use [23].

### Conclusion

Addressing today’s health care issues requires well-educated and competent new generations of medical professionals to be good researchers, lifelong learners, and excellent clinicians. This will not be achieved unless they become competent in using ICT. Therefore, preprofessional students in medical education need to be equipped with ICT knowledge and skills to enable them to use the varied informatics resources offered to them in an efficient way. Accordingly, this research study has made 3 important conclusions: (1) the ICT skills of the students before the course seemed insufficient; (2) the magnitude of the improvements to the students’ ICT knowledge and skills through the informatics course was obvious in most of the students’ performance, but the findings reveal that more practice was required; and (3) the attitudes of the students toward using e-learning using their computer skills was considered positive, although the potential of Web-based learning in medical training was not apparent to all the students. On the basis of these conclusions, several recommendations are proposed: First, the use of HSC email should be promoted among preprofessional students as a standard communication medium in the university, so emails need to be checked and responded to regularly. Second, a supplementary section should be added to the course on informatics in medicine. This should talk about the different computer systems (including digital mobile technology) and Web-based programs used in medical education and training, such as computer-assisted learning, expert systems, and surgical simulation programs. Furthermore, include statistical packages in the curriculum of the course to enable the acquisition of the required skills in data analysis and spreadsheet use, including how to use charts for the presentation of results. Third, more time should be provided for practicing different ICT skills. This requires an increase in the time spent on teaching and practicing ICT for educational purposes. This could be possible if the module of the informatics course were given more flexibility in terms of time throughout the medical education curriculum. This has also been recommended by other studies [19,24]. Finally, the medical faculty’s policy should be geared toward making the learning environment of the students electronic based to encourage the staff and students to use it, as suggested by other studies [24,25].

## Abbreviations

HSC: Health Sciences Center
ICT: information and communication technology
SPSS: Statistical Package for the Social Sciences
